# Commentary: Data Processing Thresholds for Abundance and Sparsity and Missed Biological Insights in an Untargeted Chemical Analysis of Blood Specimens for Exposomics

**DOI:** 10.3389/fpubh.2021.755837

**Published:** 2022-01-17

**Authors:** Pekka Keski-Rahkonen, Oliver Robinson, Rossella Alfano, Michelle Plusquin, Augustin Scalbert

**Affiliations:** ^1^Nutrition and Metabolism Branch, International Agency for Research on Cancer (IARC/WHO), Lyon, France; ^2^Medical Research Council Centre for Environment and Health, School of Public Health, Imperial College London, London, United Kingdom; ^3^Centre for Environmental Sciences, Hasselt University, Hasselt, Belgium

**Keywords:** metabolomics, pre-processing, data analysis, exposome, exposomics

## Introduction

We read with interest the paper by Barupal et al. on the effect of untargeted metabolomics data filtering thresholds that was recently published in (1). The authors used publicly available liquid chromatography-mass spectrometry data of 499 newborn cord blood samples. This data was generated by us in December 2015, and later published as part of our studies on the association of cord blood metabolome and birth weight (2, 3) and postnatal growth trajectories (4). Barupal et al. were critical of our decision to exclude sporadic, low-abundance information from the dataset before statistical analysis, suspecting we might have lost biologically relevant information. To study this, they pre-processed the data using their own methodology, imputed missing values and computed correlations between chromatographic peak height and birth weight for the features detected. They then assessed the effect of the filtering thresholds we had used, finding this to result in the loss of many features they found associated with birth weight, some of which they propose were linked to C19-steroid and acylcarnitine metabolism. Their conclusion was that we had missed these metabolites and thus insights into these pathways, supporting their view of using data processing thresholds for peak height and detection frequencies at minimal possible levels or entirely avoiding them.

While welcoming the idea of lowering filtering thresholds to allow deeper mining of the metabolomics data for exposome research, we found errors in the paper's interpretation of our work that we wish to correct. We would also like to further discuss the benefits and challenges associated with untargeted metabolomics data filtering.

## Discussion

Untargeted metabolomics relies on automatic algorithms to find chromatographic features in the mass spectrometric data. Several software tools exist, and while they share the same overall aim, there are marked differences in their output (5, 6). Methods for abundance measurement vary, and there are differences in detection frequency and in the amount of noise produced, especially for features at low abundance levels (7), so that filtering thresholds for these qualities are not directly transferrable. However, there were considerable methodological differences between our original work (2) and the study of Barupal et al. that we believe have led to errors in their interpretation of our results. Firstly, the pre-processing software was not the same, and different parameters for feature finding and intensity measurement were used. Secondly, methods for missing value imputation, statistical models used, and the number of features included in the analysis were different.

Barupal et al. highlighted two features they claim we missed due to the filtering applied: “*m/z* 412.3035 at 5.75 min” (speculative hydroxy-acyl carnitine) and “*m/z* 289.2162 at 4.83 min” (speculative testosterone). These features were shown to not reach the chosen threshold (chromatographic peak height >10,000 in at least 2% of the samples). However, in contrast to what the Barupal et al. paper claims, both features passed the filtering in our original study, and can be found in the published dataset (3) (available from MetaboLights). The disagreement seems to be related to differences in data pre-processing. Barupal et al. used MS-DIAL, and the highest peak in the dataset was reportedly 12,392,001, whereas in our study, based on Agilent MassHunter, the highest peak was 15,115,12. A similar relative difference was seen for the maximum peak heights of “*m/z* 289.2162 at 4.83 min” and “*m/z* 412.3035 at 5.75 min,” which in the Barupal et al. paper were 11,937 and 11,160, respectively, but 15,661 and 15,801, respectively, in our dataset.

Thus, we did not miss “*m/z* 412.3035 at 5.75 min,” which we also found associated with birthweight and identified as 3-hydroxyhexadecadienoylcarnitine (acylcarnitine C16:1) (2). We also detected “*m/z* 289.2162 at 4.83 min,” but in contrast to the unadjusted analysis of Barupal et al. it was not associated with birthweight in our model adjusted for gestational age, cohort, sex of the child, maternal height, maternal weight, and paternal height after multiple testing correction, so it was not discussed in our original paper (2). Barupal et al. suggested this feature is “probably testosterone,” but this is not correct based on the large difference in retention times when compared against testosterone reference standard (4.8 vs. 5.9 min, respectively).

**Figure 1:**
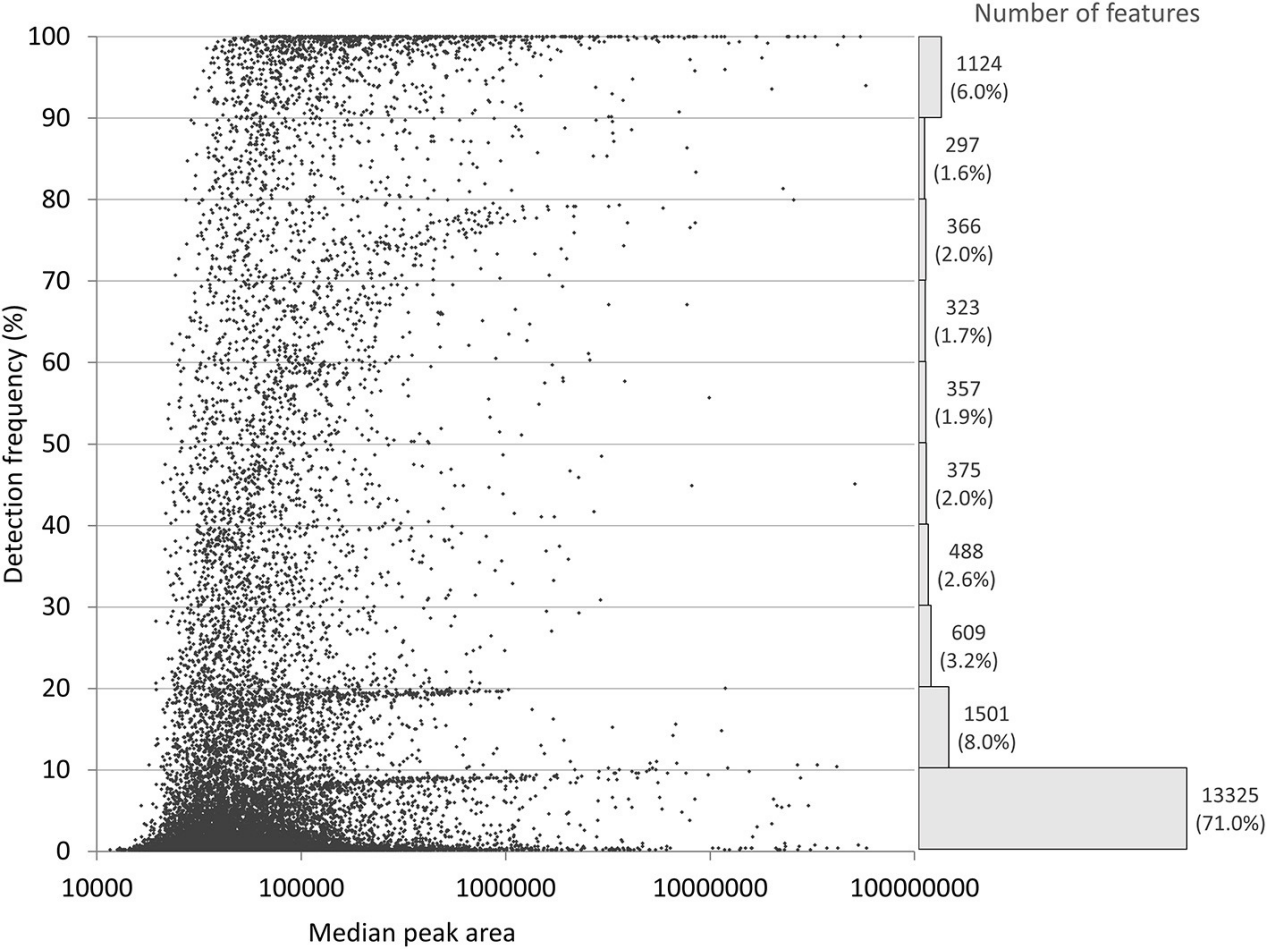
Distribution of the 18,766 features with a peak height above 10,000 in our original study, before filtering on detection frequency (2). Detection frequency refers to the percentage of samples where the feature was detected. The histogram shows the number and relative frequency of features per each 10% class.

The main conclusion of the Barupal et al. paper was that minimal or no thresholds for intensity and detection frequency should be used for metabolomics data filtering. We agree that this will minimize the loss of information. However, it will also result in a very large number of features with mostly missing values, as shown in Figure 1 that presents the discussed dataset prior to any detection frequency-based filtering. A missing value can be due to undetectably low or non-existent signal, but also related to the algorithm's inability to recognize a feature. This makes it difficult to find a universally applicable imputation strategy (8). Moreover, sensitivity of the feature finding methods leads to the presence of noise in the data, especially at low intensity levels (7). Noise and infrequent features are commonly filtered out in studies such as our original work for two main reasons: (1) analysis of extensively imputed data may lead to compromised inferences, and (2) high number variables increases the penalization of *p*-values, and therefore reduce statistical power. In our study, we intentionally filtered our data to a level we considered provided the optimal balance between metabolite detection and quality of measurements for our quantitative analysis.

Data filtering, especially on feature intensity, requires familiarity with the analytical instruments and methods used. In the Barupal et al. paper, much emphasis was based on the assumed dynamic range of the mass spectrometer and the signal-to-noise ratio (S/N) of the peaks. However, a method of extrapolating minimum usable abundance from a dynamic range estimate, or by using S/N, does not ensure analytical performance at the lowest levels (9). For example, the US EPA specifies a statistical approach to detection limits for environmental pollutants, including repeatability of the measurement rather than abundance or S/N alone (10).

For these reasons, we cannot agree with the Barupal et al. paper's suggestion that our data was “poorly explored” and that we “may have missed many metabolic hypotheses in relation to birth weight.” There are different ways to analyze the same untargeted metabolomics data and we made informed decisions on the filtering thresholds that we believe best served our statistical analyses. For other purposes and statistical models, different strategies may be better suited, and we agree that in studies where the data analysis tolerates infrequently detected features or extensively imputed data, an entirely unfiltered dataset would be valuable. For instance, these methods may lend themselves to (sufficiently powered) exploratory studies, with the metabolic feature categorized as detectable or non-detectable.

In conclusion, while we welcome the development and application of new pre-processing and filtering methods in the metabolomics field, the application of less stringent filtering thresholds by Barupal et al. did not demonstrate additional metabolic insights over our original study. The choice of pre-processing and filtering methods should consider the study design and implications on the final statistical analysis.

## Author Contributions

PK-R, OR, RA, and AS contributed to the conception of the commentary. PK-R wrote the first draft of the manuscript and produced the figure. All authors contributed to the article and approved the submitted version.

## Funding

OR was supported by a UKRI Future Leaders Fellowship (MR/S03532X/1). RA received funding from the Bijzonder Onderzoeksfonds (BOF) Hasselt University through a Ph.D. fellowship.

## Author Disclaimer

Where authors are identified as personnel of the International Agency for Research on Cancer/World Health Organization, the authors alone are responsible for the views expressed in this article and they do not necessarily represent the decisions, policy or views of the International Agency for Research on Cancer/World Health Organization.

## Conflict of Interest

The authors declare that the research was conducted in the absence of any commercial or financial relationships that could be construed as a potential conflict of interest. The handling editor declared a past collaboration with one of the authors OR.

## Publisher's Note

All claims expressed in this article are solely those of the authors and do not necessarily represent those of their affiliated organizations, or those of the publisher, the editors and the reviewers. Any product that may be evaluated in this article, or claim that may be made by its manufacturer, is not guaranteed or endorsed by the publisher.
